# Matrine induces autophagy in human neuroblastoma cells via blocking the AKT-mTOR pathway

**DOI:** 10.1007/s12032-022-01762-4

**Published:** 2022-08-16

**Authors:** Nanjing Liu, Chunmei Yang, Li Yang, Ting Li, Maoyuan Gong, Haobiao Wang, Jun Zhang, Hui Zhao, Lin Zou, Xiaoyan He

**Affiliations:** 1 Center for Clinical Molecular Medicine, Ministry of Education Key Laboratory of Child Development and Disorders, China International Science and Technology Cooperation Base of Child Development and Critical Disorders, Chongqing Key Laboratory of Pediatrics, National Clinical Research Center for Child Health and Disorders, Children’s Hospital of Chongqing Medical University, 136 Zhongshan 2nd Road, Yuzhong District, Chongqing, 400014 China; 2 Key Laboratory of Laboratory Medical Diagnostics, Ministry of Education, Chongqing Medical University, Chongqing, 400016 China; 3 Department of Oncological Surgery, Children’s Hospital of Chongqing Medical University, Chongqing, 400014 China; 4 School of Biomedical Sciences, Faculty of Medicine, The Chinese University of Hong Kong, Hong Kong, 999077 China; 5 Clinical Research Unit, Children’s Hospital of Shanghai Jiaotong University, Shanghai, 200062 China

**Keywords:** Neuroblastoma, Matrine, Cell proliferation, Autophagy

## Abstract

Neuroblastoma (NB) is one of the most common malignant solid tumors in children. Despite significant advances in the treatment strategy, the long-term survival rate of NB patients is only 50%. Developing new agents for NB patients deserves attention. Recent research indicates that matrine, a natural quinolizidine alkaloid component extracted from the traditional Chinese medicine Sophora root, is widely used for various diseases, including antitumor effects against a variety of cancers. However, the effect of matrine on NB is unknown. Herein, we found that matrine exerted antiproliferative activity in human NB cells in dose- and time-dependent manner. Matrine triggered autophagy in NB cells by blocking the AKT-mTOR signaling pathway and suppressing the phosphorylation of AKT and mTOR. 3-Methyladenine (3-MA), a PI3K inhibitor, protected against matrine-induced inhibition of cell proliferation, further supporting that the antitumor activity of matrine was at least partly autophagy-dependent. In vivo, matrine reduced tumor growth of SK-N-DZ cells in a dose-dependent manner. Matrine treatment significantly declined the phosphorylation of AKT and mTOR and enhanced the LC3 II/GAPDH ratio in NB xenografts. Altogether, our work uncovered the molecular mechanism underlying matrine-induced autophagy in NB and provided implications for matrine as a potential therapeutic agent against NB.

**Supplementary Information:**

The online version contains supplementary material available at 10.1007/s12032-022-01762-4.

## Introduction

Neuroblastoma (NB), the most common solid tumor in childhood, accounts for more than 7% of pediatric tumors and 15% of all pediatric cancer deaths [1, 2]. NB displays diverse clinical presentations ranging from spontaneous regression to aggressive progression [3]. On the basis of the International Neuroblastoma Risk Group (INRG) classification system incorporating patient age at diagnosis, INSS stage, DNA ploidy status, MYCN status, and tumor histopathology, NB patients can be classified into three risk groups (low, intermediate, and high risk) and individually treated based on stratification [4]. Current therapeutic strategies for NB patient management include observation alone, surgery, chemotherapy, radiotherapy, biotherapy, and immunotherapy. The clinical experience with NB suggests that risk-stratified therapy is beneficial, especially for low- and intermediate-risk patients with localized NB tumors and favorable clinical presentations [5, 6]. These patients tend to have an excellent overall survival probability with little or no cytotoxic therapy. However, 60% of patients have high-risk NB, with the 5-year survival rate of less than 50%, though these patients received aggressive multimodal therapies, including surgery, induction chemotherapy, radiotherapy, autologous stem cell transplantation and immunotherapy with anti-disialoganglioside (GD2) monoclonal antiboby [7]. Of concern, almost all patients treated with high-risk NB experience a series of treatment-related acute toxicities (severe transient myelosuppression, chemotherapy-induced renal dysfunction, poor weight gain, etc.) and late effects (secondary malignancy, endocrinopathy, renal dysfunction, hearing loss, growth failure, etc.) [8]. Given the poor outcome and the burden of toxicities of high-risk NB patients, innovative therapeutics with little toxicity or few side effects are urgently needed.

Some natural products, such as traditional Chinese medicine, are applied as alternative treatments for cancer because of their wide biological activity and relatively low toxicity. Matrine, a natural quinolizidine alkaloid, is an effective monomer extracted from *Sophora flavescens Ait*, *Sophora subprostrata* or *Sophora alopecuroides* in traditional Chinese medicine. Recent evidence has indicated that matrine exerts antitumor effects on various tumors, including lung cancer [9], breast cancer [10], gastric cancer [11], leukemia [12] and pancreatic cancer [13]. For example, matrine inhibited growth in human ovarian cancer A2780 and SKOV3 cells in a dose- and time-dependent manner [14], exhibited antitumor effects against human hepatoma G2 cells by inducing apoptosis [15], and suppressed the proliferation, migration and invasion of human prostate cancer DU145 and PC-3 cells in a dose- and time-dependent manner [16]. However, the antitumor effects of matrine on NB cells and its underlying molecular mechanisms are not yet clear.

Here, we investigated the antitumor effects of matrine on human NB SK-N-AS and SK-N-DZ cells and its underlying mechanism and found that matrine inhibited the proliferation of NB cells in vitro and in vivo by inducing autophagy by blocking the AKT (AKT serine/threonine kinase)-mTOR pathway. These results support the potential therapeutic utility of matrine-mediated autophagy activation and a new role for NB treatment.

## Materials and methods

### Cell culture

The human NB cell lines SK-N-AS (ATCC® CRL-2137™) and SK-N-DZ (ATCC® CRL2149™) were obtained from the American Type Culture Collection (ATCC, Rockville, MA, USA). Mycoplasma testing was performed using a Mycoplasma Detection kit (Beijing Solarbio Science & Technology Co., Ltd.). SK-N-AS and SK-N-DZ cells were maintained in Dulbecco’s modified Eagle’s medium (Gibco; Thermo Fisher Scientific, Inc.) supplemented with 10% fetal bovine serum (FBS) and with 1% penicillin and streptomycin antibiotics.

### CCK-8 assay

The short-term effects of matrine (#S2322, Selleck, USA) on cell metabolic activity were assessed using the CCK-8 assay. Cells were seeded at a density of 5000 cells/well in a 96-well plate and treated with matrine at the indicated concentrations (0, 0.2, 0.4, 1, 2, 4, and 6 mM) for 24, 48 or 72 h. CCK-8 solution (#KQ749, DOJINDO, Japan) was added (10 μl/well), and plates were incubated for 4 h at 37 °C. The plates were read on a microplate system (Gen5 GHS 2.0, USA) at 540 nm. Assays were performed in triplicate in 3 independent experiments.

### Colony-formation assay

SK-N-AS and SK-N-DZ cells at the exponential growth phase were trypsinized and plated in triplicate wells in a 6-well plate (500 cells/well). After 12 h of incubation, cells were treated with DMSO or the indicated IC50 concentrations of matrine in the absence or presence of 3-MA (2 mM, #S2767, selleck, USA). The cells were treated for seven days, washed, and stained with 0.05% w: v crystal violet. And the colonies > 100 μm in diameter were counted.

### Western blot

Subconfluent cells were washed with PBS and lysed using RIPA buffer containing 1 × protease inhibitor cocktail and phosphatase inhibitors. Equal amounts of protein samples (20 μg/lane) were fractionated by SDS–polyacrylamide gel electrophoresis and transferred onto a polyvinylidene difluoride (PVDF) membrane (Millipore, USA) in 25 mM Tris and 192 mM glycine. Membranes were blocked with 5% nonfat dry milk in TBS and 0.05% Tween-20 for 2 h and then probed with the indicated antibodies at desired dilutions at 4 °C overnight, followed by corresponding secondary antibodies. The results of WB analyses were quantified using ImageJ software. The antibodies used in WB were as follows: anti-LC3 (1:1000, #4108, CST, USA), anti-GAPDH (1:5000, #10494-1-AP, Proteintech, USA), anti-p-S2448-mTOR (1:1000, #2971, CST, USA), anti-mTOR (1:1000, #2972, CST, USA), anti-p-S473-AKT (1:1000, #4060p, CST, USA), and anti-AKT (1:1000, #9272s, CST, USA).

### Transmission electron microscopy

SK-N-AS and SK-N-DZ cells were exposed to matrine for 48 h and fixed with 2.5% glutaraldehyde solution overnight, followed by fixation with 1% OsO4. After dehydration in graded ethanol, 10 nm thin sections were sliced and stained with 2% uranyl acetate. Observation was performed on a transmission electron microscope (TEM, Hitachi, S-3000 N, Japan). High-resolution digital images were acquired from 5 randomly selected fields for samples of each condition.

### Confocal immunofluorescence microscopy

Cells grown in glass coverslips were treated with or without matrine for 48 h, rinsed in PBS, fixed with 4% formaldehyde for 20 min and then permeabilized with 0.3% Triton X-100. After rinsing in PBS, cells were probed with LC3 antibody (1:500, #4108, CST, USA) at 4 °C overnight. After being washed three times with PBST, cells were incubated with Fluor Cy3-conjugated secondary antibodies (1:100, #SA00009-2, Proteintech, USA) for 1 h at room temperature. The nuclei were stained with DAPI for 10 min before being mounted with aqueous mounting medium and then visualized using a Leica laser confocal microscope (C2 + system, Nikon, Japan).

### Flow cytometry analysis of apoptosis and cell cycle

SK-N-AS and SK-N-DZ cells were seeded into 6-well plates and treated with the indicated concentration of matrine for 48 h. For apoptosis analysis, cells were harvested by treatment with trypsin, washed twice with cold PBS and resuspended in 100 μl of Annexin V binding buffer. Cells were then stained with Annexin V labeled by FITC and PI for 20 min at 37 °C in the dark. Within one hour, the fluorescence intensity of the early and late apoptotic cells were detected by using FACS Calibur flow cytometer (BD Biosciences, USA). For cell cycle assay, cells were washed with PBS, and then trypsinized, washed with PBS twice and fixed in 70% ethanol at 4 °C. After fixation, the ethanol was removed, and the PI-staining solution was added. Then cells were incubated for 30 min. Fluorescence signals were analyzed immediately using a FACS Calibur flow cytometer (BD Biosciences, USA). Dot plots and histograms were analyzed by FlowJo software.

### In vivo xenograft model

Female BALB/c nude mice, aged 4–6 weeks, weighing 18–20 g, were fed in a barrier facility, and the experiments were approved by the Chongqing Medical University Institutional Animal Care and Use Committee and the Animal Ethics Committee. To investigate the suppressive effect of matrine on tumor development in vivo, each mouse was injected subcutaneously with 5 × 10^6^ SK-N-DZ cells (100 μl in PBS) into the right flank. Once a palpable tumor was present, mice were treated intraperitoneally (IP) with either vehicle or matrine (50, 75, 100 mg/kg) every three days. Mice were sacrificed 30 days after transplanting and the tumors were dissected out. Tumor size was measured using a caliper and calculated using the following formula: Volume = 1/2 × (length × width^2^), and tumors were weighed and collected for further analysis.

### Immunohistochemistry

Formalin-fixed and paraffin-embedded tissue sections were deparaffinized in xylene and graded alcohols, hydrated, and washed in a PBS solution. After antigen retrieval in a sodium citrate buffer (pH6) in a microwave oven, the endogenous peroxidase was blocked by 0.3% H_2_O_2_ for 20 min. Sections were incubated overnight at 4 °C with primary antibodies anti-LC3 (1:400, #4599, CST, USA) and anti-Ki67 (1:400, #9027, CST, USA), washed with PBS and subsequently incubated with the biotin-streptavidin horseradish peroxidase labeled Goat anti-rabbit IgG (#SP-9001, Beijing Zhongshan Jinqiao Biotechnology Co., Ltd.) for 1 h at room temperature. Signals were visualized using a peroxidase substrate and hematoxylin counterstaining for 1 min at room temperature.

### Statistical analysis

All data present as mean ± SEM using Prism 6.0 (GraphPad Software). Statistically significant was analyzed using unpaired two-tailed Student’s *t*-test or one-way ANOVA. Data were deemed to be statistically significant if *P* < 0.05.

## Results

### Matrine inhibited the cell growth of NB cells in vitro

**Fig. 1:**
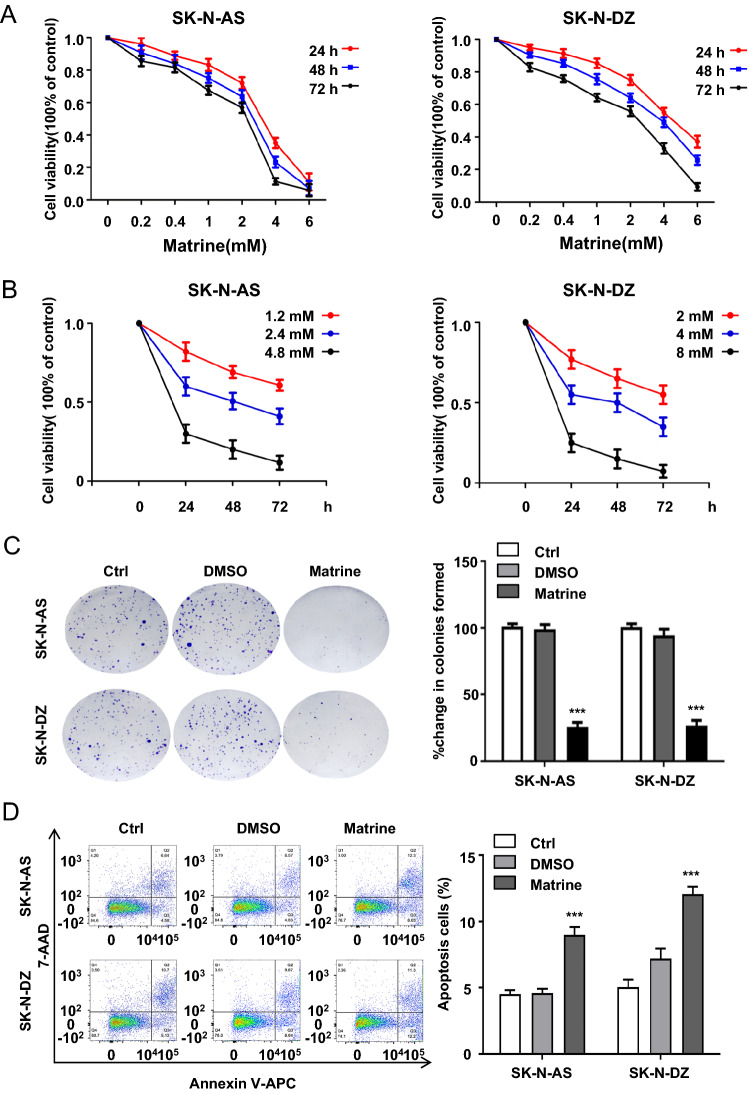
Cell growth was suppressed by matrine in human NB cells in vitro. **A** The effect of matrine on the cytotoxicity of the SK-N-AS and SK-N-DZ cells by CCK-8 assay. **B** The effect of matrine at different concentrations on NB cell proliferation by CCK-8 assay. **C** The effect of matrine on the proliferation of NB cells by colony formation assay. **D** The effect of matrine on the apoptosis of NB cells by flow cytometry. Data were presented as the mean ± standard deviation. ***P < 0.001 *vs.* control

To determine the effect of matrine (Fig. S1) on the proliferation of human NB cells, SK-N-AS and SK-N-DZ cells were examined by CCK-8 assay after matrine treatment. The proliferation of SK-N-AS and SK-N-DZ cells was significantly inhibited by matrine treatment in a dose- and time-dependent manner (Fig. 1A, B). The IC50 values of matrine on SK-N-AS and SK-N-SZ cells at 48 h were 2.4 and 4 mM, respectively, which were appropriately used for further study. To evaluate the effect of matrine on cellular viability, a colony formation assay was performed. SK-N-AS and SK-N-DZ cells were markedly decreased after incubation with matrine compared with those in the control group (*P* < 0.001) (Fig. 1C). To examine whether cell growth inhibition is related to the cell cycle arrest of NB cells, we measured the role of matrine in cell cycle distribution. However, no significant change in cell cycle distribution was found in either SK-N-AS or SK-N-DZ cells (*P* > 0.05) (Fig. S2). We then detected the apoptosis rate by flow cytometry analysis with annexin V/7-AAD double staining in SK-N-AS and SK-N-DZ cells. Compared with the control group, the ratio of apoptotic cells (Ann + /PI-), significantly increased in the matrine group after 48 h of treatment (*P* < 0.001) (Fig. 1D).

### Matrine triggered autophagy in NB cells

**Fig. 2:**
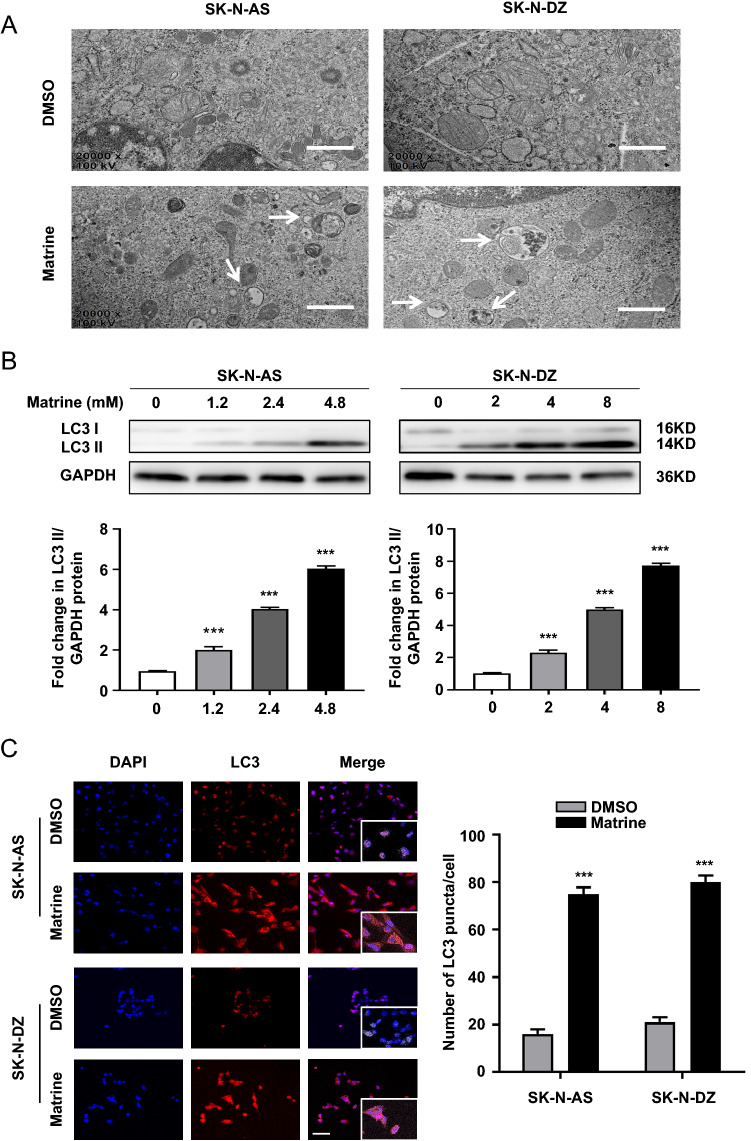
Autophagy was induced by matrine in human NB cells. **A** Matrine induced the increase of NB cell autophagosome by transmission electron microscopy (× 20,000, scale bar = 1 μm). **B** The effect of matrine on the protein level of LC3 in the SK-N-AS and SK-N-DZ cells by western blot. **C** The expression of LC3 in SK-N-AS and SK-N-DZ cells treated with matrine by immunofluorescence staining (× 400, scale bar = 50 μm). Data were presented as the mean ± standard deviation. **P < 0.01 and ***P < 0.001 *vs.* control

The autophagy pathway has broad implications in many physiological and pathological processes, including tumorigenesis. Recent findings have revealed that autophagy induced by chemotherapeutic agents may have a suppressive role in cancer [17, 18]. To determine whether matrine affects autophagy in NB cells, we visualized the induction of autophagy using transmission electron microscopy. The number of autophagosomes markedly increased in SK-N-AS or SK-N-DZ cells treated with matrine after 48 h, but not in the control group (Fig. 2A). Next, we investigated the effect of matrine on the formation of autophagosomes in vitro by detecting the conversion of LC3 I to lipidated LC3 II, a classical marker of autophagosome formation. Given that LC3 II/GAPDH is better indicator of autophagosome formation than LC3 II/LC3 I [19], as shown in Fig. 2B, we found that matrine caused the induction of autophagy, as evidenced by increased LC3 II/GAPDH, in a dose-dependent manner in both SK-N-AS and SK-N-DZ cells (*P* < 0.001). We then detected the distribution of endogenous LC3 puncta in SK-N-AS and SK-N-DZ cells by immunofluorescence. Significant increase of LC3 puncta were found in both SK-N-AS and SK-N-DZ cells treated with matrine after 48 h (*P* < 0.001) (Fig. 2C), indicating that matrine triggered autophagy in NB cells.

### Matrine induced autophagic flux in NB cells

**Fig. 3:**
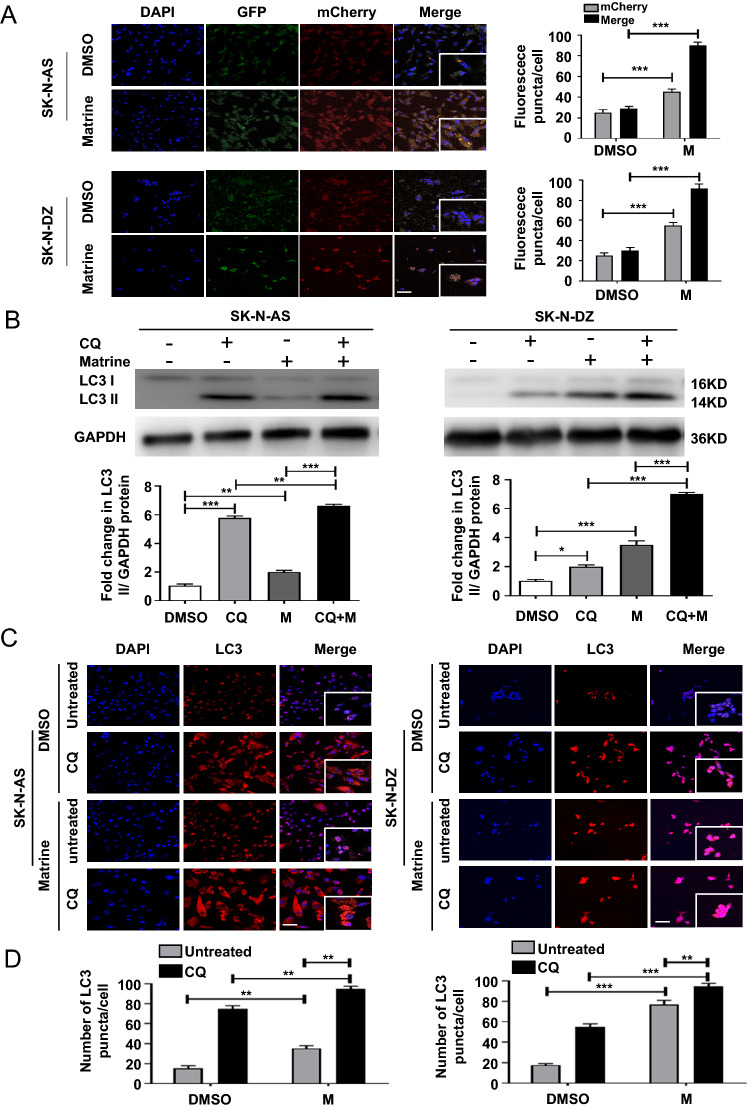
Autophagic flux was enhanced by matrine in human NB cells. **A** Autophagy flux was detected in SK-N-AS and SK-N-DZ cells treated with matrine by confocal laser microscopy (× 400, scale bar = 50 μm) (left). Quantification of the number of fluorescent puncta exhibiting GFP or mCherry fluorescence per cell (right). **B** The expression of LC3 in the SK-N-AS and SK-N-DZ cells treated by matrine or in combination with chloroquine by western blot. **C** The expression of LC3 in SK-N-AS and SK-N-DZ cells treated with matrine in the presence or absence of chloroquine by immunofluorescence staining (× 400, scale bar = 50 μm). **D** Quantitative analysis of LC3 spots per cell. Data were presented as the mean ± standard deviation. *P < 0.05, **P < 0.01, and ***P < 0.001 *vs.* control. *M* matrine, *CQ* chloroquine

Both autophagy inducers and inhibitors cause build-ups of LC3 II and LC3-positive puncta. To further distinguish the role of matrine in autophagy, we observed the impact on autophagic flux using tandem monomeric GFP-mCherry-tagged LC3. We found increased formation of autophagosomes (yellow fluorescence) and autolysosomes (red fluorescence) in both SK-N-AS and SK-N-DZ cells treated with matrine after 48 h (*P* < 0.001) (Fig. 3A). We then examined matrine-induced autophagic flux in the presence or absence of chloroquine (CQ), an autophagosome-lysosome fusion inhibitor. Compared with matrine alone, an enhanced LC3 II/GAPDH ratio was observed in both SK-N-AS cells (*P* < 0.001) and SK-N-DZ cells (*P* < 0.001) treated with matrine combined with CQ (Fig. 3B). Also, when compared to the cells treated with CQ, significant difference of the LC3 II/GAPDH ratio were also found in both SK-N-AS cells(*P* < 0.01) and SK-N-DZ cells (*P* < 0.001) treated with matrine combined with CQ (Fig. 3B). Next, we confirmed this observation by detecting the numbers of LC3 puncta in both SK-N-AS and SK-N-DZ cells treated with matrine combined with CQ compared to matrine alone or CQ alone(*P* < 0.01) (Fig. 3C and D). Taken together, these data indicated that matrine functions as an autophagy inducer in NB cells.

### Matrine induced autophagy even in the presence of the autophagy inhibitor 3-MA

**Fig. 4:**
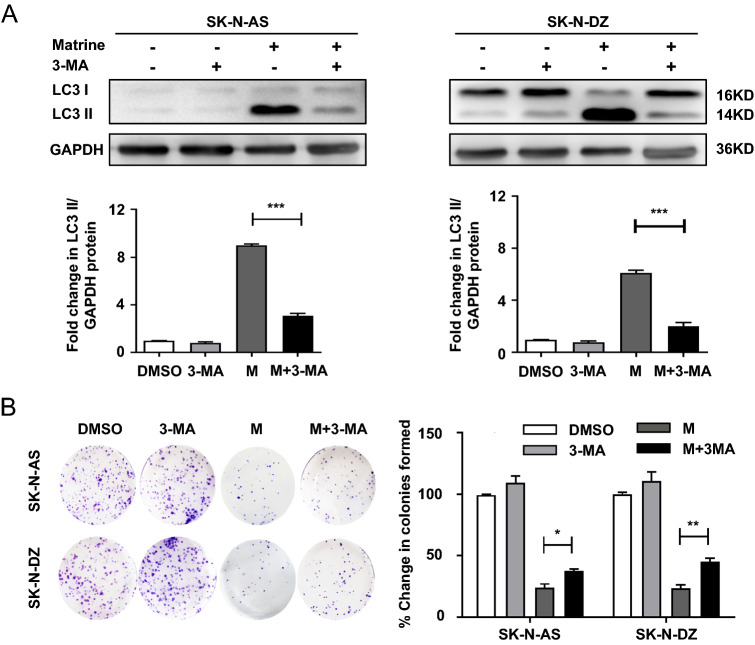
Matrine induced autophagy in NB cells in the presence of the autophagy inhibitor 3-MA. **A** The expression of LC3 was detected in SK-N-AS and SK-N-DZ cells treated with matrine alone or in combination with 3-methyladenine by western blot. **B** The effects of matrine alone or in combination with 3-MA on the proliferation of NB cells by colony formation assay. Data were presented as the mean ± standard deviation. *P < 0.05, **P < 0.01, and ***P < 0.001 *vs.* control. *M* matrine, *CQ* chloroquine, *3-MA*, 3-methyladenine

To further determine whether the anticancer effect of matrine in NB cells occurred through the induction of autophagy, cells were treated in the presence or absence of the autophagy inhibitor 3-MA, a class III phosphatidylinositol 3-kinase inhibitor that prevents the formation of autophagosomes [20], followed by treatment with matrine. The combination of 3-MA with matrine significantly decreased the LC3 II/GAPDH ratio (*P* < 0.001) (Fig. 4A). 3-MA also rescued cell proliferation from matrine-induced antiproliferative activity, as demonstrated by clonogenic assay in both SK-N-AS cells (*P* < 0.05) and SK-N-DZ cells (*P* < 0.01) (Fig. 4B). Similar results were obtained by the CCK8 test in SK-N-AS cells (*P* < 0.01) and SK-N-DZ cells (*P* < 0.001) (Fig. S3) These results suggested that inhibition of autophagy counteracted the inhibitory effect of matrine in NB cells.

### Matrine inhibited the phosphorylation of AKT and mTOR

**Fig. 5:**
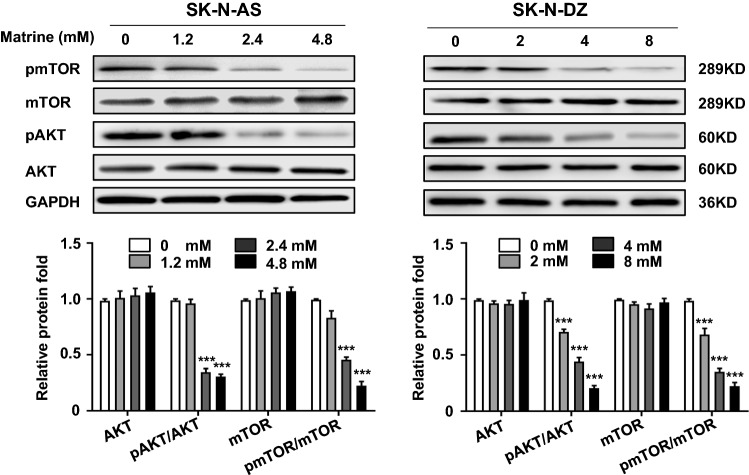
Matrine inhibited the activity of AKT-mTOR signaling pathway in NB cells. The effect of matrine on the protein level of AKT, p-S473-AKT, mTOR, p-S2448-mTOR by western blot. Data were presented as the mean ± standard deviation. ***P < 0.001 *vs.* control

The AKT-mTOR pathway is a well-established pathway as a key negative regulator of autophagy and is frequently activated in cancer cells [21]. Hence, we examined the role of the AKT-mTOR pathway in matrine-treated SK-N-AS and SK-N-DZ cells. The results showed that the expression levels of AKT and mTOR were not altered, but the phosphorylation levels of AKT and mTOR were significantly inhibited by matrine in a dose-dependent manner in SK-N-AS or SK-N-DZ cells (*P* < 0.001) (Fig. 5).

### Matrine-induced autophagy alleviated NB burden in vivo

**Fig. 6:**
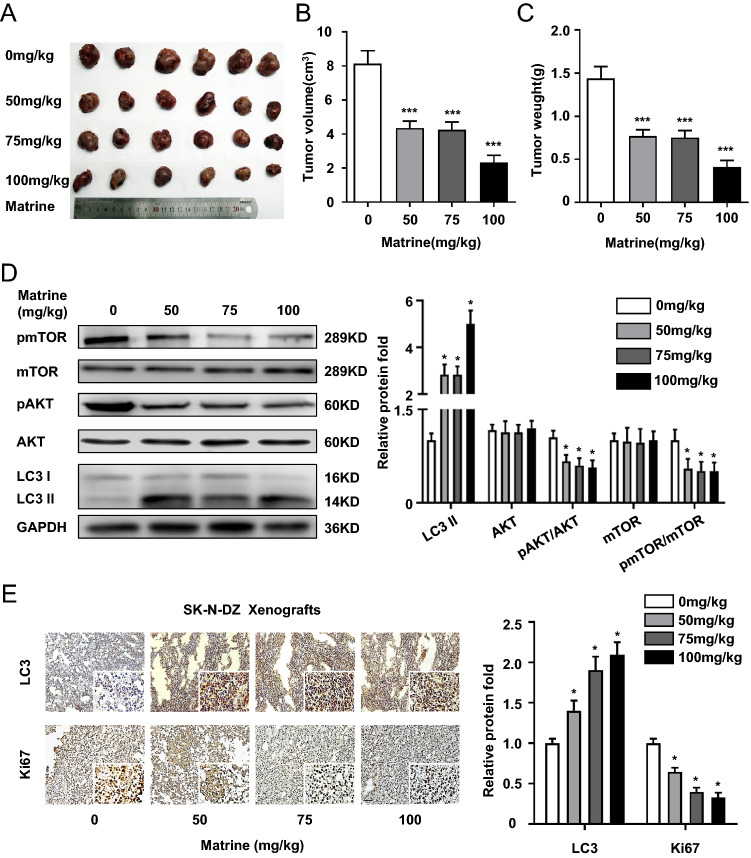
Matrine inhibited tumor growth of SK-N-DZ cells in vivo. **A** At the end of the experiments, SK-N-DZ xenografted tumors exposed to different concentration of matrine were presented. **B** The tumor volumes of nude mice in the xenografted tumors. **C** The tumor weight of nude mice in the xenografted tumors. **D** The protein level of LC3, AKT, p-S473-AKT, mTOR, p-S2448-mTOR of xenografts by western blot assay. **E** The expression levels of LC3 and Ki67 of xenografts by HE staining (× 200, scale bar = 100 μm). Data were presented as the mean ± standard deviation. *P < 0.05, **P < 0.01, and ***P < 0.001 *vs.* control

To further investigate the in vivo efficacy of matrine in NB, we employed NB models by implanting SK-N-DZ cells subcutaneously into nude mice. After each mouse subcutaneously with 5 × 10^6^ SK-N-DZ cells (100 μl in PBS) into the right flank, most of them develop a palpable tumor around 5–7 days with a tumor rate of 100%. No significant difference was found. The tumor volumes in the 0, 50, 75 and 100 mg/kg groups were 8.145 ± 0.754, 4.364 ± 0.398, 4.258 ± 0.459 and 2.326 ± 0.420 cm^3^, respectively, after inoculation of cells for 30 days. Tumor xenografts from mice treated with different concentration of matrine grew at a slower rate than those from mice treated with vehicle, as revealed by the reduced tumor volumes and weights (*P* < 0.001) (Fig. 6A–C). However, there was no significant difference among the three matrine-treated groups. Furthermore, matrine significantly reduced the protein expression levels of p-AKT and p-mTOR and enhanced the LC3 II/GAPDH ratio in the tumors, consistent with the results obtained from the analysis of NB cells in vitro (*P* < 0.05) (Fig. 6D). We then immunohistochemically stained tumor tissues from SK-N-DZ xenograft models with antibodies of LC3 and Ki67, which is widely used as a proliferation marker in human tumor cells [22]. The results also showed that matrine increased the expression level of LC3 and decreased the expression level of Ki67 (*P* < 0.05) (Fig. 6E). These results indicated that matrine suppressed the growth of NB in vivo by inhibiting autophagy and PI3K/AKT/mTOR signaling.

## Discussion

The present study indicated that matrine induced marked growth suppression and autophagy in NB cell lines in vitro and subcutaneous xenograft of SK-N-DZ cells in vivo. Furthermore, we demonstrated the mechanism by which matrine triggered autophagy in NB cells by blocking the AKT-mTOR pathway. In addition, we found that 3-MA significantly protected matrine-induced cell viability inhibition, further supporting that matrine-induced inhibition of NB cell proliferation was autophagy-dependent. Thus, to our knowledge, this is the first study to describe the effect of matrine on NB cells in vitro and in vivo.

Autophagy is an evolutionarily conserved stress response and degradative mechanism involved in the degradation of damaged or dysfunctional intracellular components delivered by double-membrane autophagosomes to lysosomes [23, 24]. Dysfunctional autophagy has been shown to contribute to carcinogenesis [25]. Notably, according to distinct cell conditions and cancer cell subtypes, the consequences of autophagy activation will be very different in response to chemotherapy drugs, which may enhance or weaken drug efficacy [26]. On the one hand, autophagy acts as a drug-resistant mechanism to promote the survival of cancer cells by removing damaged organelles and recycling nutrients under chemotherapy stress. In this case, the main therapeutic strategy is to perturb autophagy to improve drug sensitivity [27]. On the other hand, autophagy induced by chemotherapeutic agents exerts antitumor effects by two different functional forms of autophagy, namely, “cytotoxic function” leading to autophagy cell death alone or by apoptosis induction and “cytostatic function” leading to cell growth inhibition in an apoptosis-independent manner [28]. Interestingly, both autophagy inducers and inhibitors have a therapeutic advantage in cancer therapy. Autophagy inhibitors (e.g., chloroquine (CQ) or hydroxychloroquine (HCQ)) [29–31] and autophagy inducers (e.g., ivermectin, or rapamycin) [32, 33] have shown promising antitumor effects. This highlights the importance of understanding the roles of autophagy to further provide new opportunities for therapeutic strategies.

Our study indicated that matrine inhibited the growth of NB cells in a dose- and time-dependent manner. Notably, matrine markedly increased autophagy and autophagic flux in human NB SK-N-AS and SK-N-DZ cells in the presence of the autophagy inhibitor CQ, suggesting that matrine functions as an autophagy inducer and thereby represents a feasible strategy for the treatment of NB tumors. Interestingly, we observed matrine-induced apoptosis in NB cells, consistent with other reports [14, 33, 34]. However, the complex relationship between autophagy and apoptosis needs further investigation.

It has been well established that the phosphoinositide 3-kinase (PI3K)-AKT-mammalian target of rapamycin (mTOR) pathway is a negative regulator of autophagy [35, 36]. AKT is a serine-threonine kinase located downstream of PI3K that activates the kinase mTOR, leading to the suppression of autophagy. Notably, the pathway plays a critical role in the pathogenesis and progression of NB tumors [37]. Pathological activation of the PI3K-AKT-mTOR pathway also frequently occurs in NB and correlates with poor prognosis [38]. PI3K-AKT-mTOR pathway inhibition severely suppresses NB cell growth in vitro and in vivo [39–41]. PI3K pathway-targeted therapies have shown preclinical promise [42].

In the present study, we found that matrine suppressed the phosphorylation of AKT and mTOR and triggered autophagy, and matrine-induced growth inhibition was rescued while preventing formation of autophagosomes with 3-MA, a class III phosphatidylinositol 3-kinase inhibitor. Our data indicated that matrine inhibited the proliferation of NB cells at least partly in an autophagy-dependent manner. Similar to other results, matrine induced autophagy via suppression of PI3K/AKT in human lung adenocarcinoma cells [34], glioma cells [33] and ovarian cancer cells [14]. These findings may have implications for matrine as an inhibitor of the PI3K-AKT-mTOR pathway for antitumor therapies.

There were several limitations in the present study. Firstly, our results lacked of data other than cell lines. Matrine-induced autophagy was only found in human NB SK-N-AS and SK-N-DZ cells, and more work is needed to confirm these findings in other NB cell lines and animal models. Secondly, the phosphorylation levels of AKT and mTOR were significantly inhibited by matrine in a dose-dependent manner, but no significant change of AKT and mTOR expression was found between the matrine treatment group and control group. The mechanism by which matrine inhibited phosphorylation of AKT-mTOR pathway has yet to be further investigated.

## Conclusion

Taken together, matrine exerts a significant antitumor effect on NB cells in vitro and in vivo. The anticancer activity of matrine was at least partly attributed to its inhibition of proliferation and autophagy induction of NB cells through blocking the PI3K/AKT/mTOR pathway. Our data provide a molecular basis for the antitumor effect of matrine in NB cells, supporting that matrine as an autophagy inducer may become a novel treatment option for NB therapy.

## Supplementary Information

Below is the link to the electronic supplementary material.

Supplementary file1 Fig. S1 The structure of matrine. Formula: C15H24N2O, Molecular Weight: 248.36, CAS No. 519-02-8 (PPT 137 kb)

Supplementary file2 Fig. S2 Effects of matrine on the cell cycle in human NB cells. Representative flow cytometry analysis of the cell cycle in SK-N-AS and SK-N-DZ cells treated with DMSO or matrine for 48 h (PPT 192 kb)

Supplementary file3 Fig. S3 3-methyladenine rescued the short-term cell viability from matrine-induced antiproliferative activity in NB cells. The short-term cell viability of SK-N-AS and SK-N-DZ cells treated with DMSO or matrine alone or in combination with 3-methyladenine were evaluated by CCK-8 assay. 3-MA, 3-methyladenine. **P<0.01, and ***P<0.001 vs. control (PPT 164 kb)

## References

[1] Maris JM (2010). Recent advances in neuroblastoma. N Engl J Med.

[2] Brodeur GM (2003). Neuroblastoma: biological insights into a clinical enigma. Nat Rev Cancer.

[3] Matthay KK, Maris JM, Schleiermacher G, Nakagawara A, Mackall CL, Diller L, Weiss WA (2016). Neuroblastoma. Nat Rev Disease Primers.

[4] Maris JM, Hogarty MD, Bagatell R, Cohn SL (2007). Neuroblastoma. Lancet (London, England).

[5] Baker DL, Schmidt ML, Cohn SL, Maris JM, London WB, Buxton A, Stram D, Castleberry RP, Shimada H, Sandler A, Shamberger RC, Look AT, Reynolds CP, Seeger RC, Matthay KK (2010). Outcome after reduced chemotherapy for intermediate-risk neuroblastoma. N Engl J Med..

[6] Strother DR, London WB, Schmidt ML, Brodeur GM, Shimada H, Thorner P, Collins MH, Tagge E, Adkins S, Reynolds CP, Murray K, Lavey RS, Matthay KK, Castleberry R, Maris JM, Cohn SL (2012). Outcome after surgery alone or with restricted use of chemotherapy for patients with low-risk neuroblastoma: results of Children's Oncology Group study P9641. J Clinical Oncol.

[7] Zafar A, Wang W, Liu G, Wang X, Xian W, McKeon F, Foster J, Zhou J, Zhang R (2021). Molecular targeting therapies for neuroblastoma: Progress and challenges. Med Res Rev.

[8] Whittle SB, Smith V, Doherty E, Zhao S, McCarty S, Zage PE (2017). Overview and recent advances in the treatment of neuroblastoma. Expert Rev Anticancer Ther.

[9] Zhang Y, Zhang H, Yu P, Liu Q, Liu K, Duan H, Luan G, Yagasaki K, Zhang G (2009). Effects of matrine against the growth of human lung cancer and hepatoma cells as well as lung cancer cell migration. Cytotechnology.

[10] Li LQ, Li XL, Wang L, Du WJ, Guo R, Liang HH, Liu X, Liang DS, Lu YJ, Shan HL, Jiang HC (2012). Matrine inhibits breast cancer growth via miR-21/PTEN/Akt pathway in MCF-7 cells. Cellular Physiol Biochem.

[11] Zhang J, Li Y, Chen X, Liu T, Chen Y, He W, Zhang Q, Liu S (2011). Autophagy is involved in anticancer effects of matrine on SGC-7901 human gastric cancer cells. Oncol Rep.

[12] Zhang S, Zhang Y, Zhuang Y, Wang J, Ye J, Zhang S, Wu J, Yu K, Han Y (2012). Matrine induces apoptosis in human acute myeloid leukemia cells via the mitochondrial pathway and Akt inactivation. PLoS ONE.

[13] Chen H, Zhang J, Luo J, Lai F, Wang Z, Tong H, Lu D, Bu H, Zhang R, Lin S (2013). Antiangiogenic effects of oxymatrine on pancreatic cancer by inhibition of the NF-κB-mediated VEGF signaling pathway. Oncol Rep.

[14] Zhang X, Hou G, Liu A, Xu H, Guan Y, Wu Y, Deng J, Cao X (2019). Matrine inhibits the development and progression of ovarian cancer by repressing cancer associated phosphorylation signaling pathways. Cell Death Dis.

[15] Zhang JQ, Li YM, Liu T, He WT, Chen YT, Chen XH, Li X, Zhou WC, Yi JF, Ren ZJ (2010). Antitumor effect of matrine in human hepatoma G2 cells by inducing apoptosis and autophagy. World J Gastroenterol.

[16] Li Q, Lai Y, Wang C, Xu G, He Z, Shang X, Sun Y, Zhang F, Liu L, Huang H (2016). Matrine inhibits the proliferation, invasion and migration of castration-resistant prostate cancer cells through regulation of the NF-κB signaling pathway. Oncol Rep.

[17] Chang CH, Bijian K, Wernic D, Su J, da Silva SD, Yu H, Qiu D, Asslan M, Alaoui-Jamali MA (2019). A novel orally available seleno-purine molecule suppresses triple-negative breast cancer cell proliferation and progression to metastasis by inducing cytostatic autophagy. Autophagy.

[18] Gewirtz DA (2014). The four faces of autophagy: implications for cancer therapy. Can Res.

[19] Klionsky DJ, Abdel-Aziz AK, Abdelfatah S (2021). Guidelines for the use and interpretation of assays for monitoring autophagy. Autophagy.

[20] Wu Y, Wang X, Guo H, Zhang B, Zhang XB, Shi ZJ, Yu L (2013). Synthesis and screening of 3-MA derivatives for autophagy inhibitors. Autophagy.

[21] Schmelzle T, Hall MN (2000). TOR, a central controller of cell growth. Cell.

[22] Sun X, Kaufman PD (2018). Ki-67: more than a proliferation marker. Chromosoma.

[23] Kondo Y, Kanzawa T, Sawaya R, Kondo S (2005). The role of autophagy in cancer development and response to therapy. Nat Rev Cancer.

[24] Parzych KR, Klionsky DJ (2014). An overview of autophagy: morphology, mechanism, and regulation. Antioxid Redox Signal.

[25] White E, Mehnert JM, Chan CS (2015). Autophagy, metabolism, and cancer. Clinical Cancer Res.

[26] Amaravadi RK, Kimmelman AC, Debnath J (2019). Targeting autophagy in cancer: recent advances and future directions. Cancer Discov.

[27] Katayama M, Kawaguchi T, Berger MS, Pieper RO (2007). DNA damaging agent-induced autophagy produces a cytoprotective adenosine triphosphate surge in malignant glioma cells. Cell Death Differ.

[28] Liu R, Li J, Zhang T, Zou L, Chen Y, Wang K, Lei Y, Yuan K, Li Y, Lan J, Cheng L, Xie N, Xiang R, Nice EC, Huang C, Wei Y (2014). Itraconazole suppresses the growth of glioblastoma through induction of autophagy: involvement of abnormal cholesterol trafficking. Autophagy.

[29] Chude CI, Amaravadi RK (2017). Targeting autophagy in cancer: update on clinical trials and novel inhibitors. Int J Mol Sci.

[30] Qureshi-Baig K, Kuhn D, Viry E, Pozdeev VI, Schmitz M, Rodriguez F, Ullmann P, Koncina E, Nurmik M, Frasquilho S, Nazarov PV, Zuegel N, Boulmont M, Karapetyan Y, Antunes L, Val D, Mittelbronn M, Janji B, Haan S, Letellier E (2020). Hypoxia-induced autophagy drives colorectal cancer initiation and progression by activating the PRKC/PKC-EZR (ezrin) pathway. Autophagy.

[31] Rebecca VW, Nicastri MC, Fennelly C, Chude CI, Barber-Rotenberg JS, Ronghe A, McAfee Q, McLaughlin NP, Zhang G, Goldman AR, Ojha R, Piao S, Noguera-Ortega E, Martorella A, Alicea GM, Lee JJ, Schuchter LM, Xu X, Herlyn M, Marmorstein R, Amaravadi RK (2019). PPT1 promotes tumor growth and is the molecular target of chloroquine derivatives in cancer. Cancer Discov.

[32] Dou Q, Chen HN, Wang K, Yuan K, Lei Y, Li K, Lan J, Chen Y, Huang Z, Xie N, Zhang L, Xiang R, Nice EC, Wei Y, Huang C (2016). Ivermectin induces cytostatic autophagy by blocking the PAK1/Akt axis in breast cancer. Can Res.

[33] Cao C, Subhawong T, Albert JM, Kim KW, Geng L, Sekhar KR, Gi YJ, Lu B (2006). Inhibition of mammalian target of rapamycin or apoptotic pathway induces autophagy and radiosensitizes PTEN null prostate cancer cells. Can Res.

[34] Wan Q, Du Z, Fang Z, Cheng H, Li C, Zhou X (2020). Matrine induces apoptosis and autophagy in human lung adenocarcinoma cells via upregulation of Cavin3 and suppression of PI3K/AKT pathway. J BUON.

[35] Xu Z, Han X, Ou D, Liu T, Li Z, Jiang G, Liu J, Zhang J (2020). Targeting PI3K/AKT/mTOR-mediated autophagy for tumor therapy. Appl Microbiol Biotechnol.

[36] Yu X, Long YC, Shen HM (2015). Differential regulatory functions of three classes of phosphatidylinositol and phosphoinositide 3-kinases in autophagy. Autophagy.

[37] Engelman JA (2009). Targeting PI3K signalling in cancer: opportunities, challenges and limitations. Nat Rev Cancer.

[38] Opel D, Poremba C, Simon T, Debatin KM, Fulda S (2007). Activation of Akt predicts poor outcome in neuroblastoma. Can Res.

[39] Opel D, Naumann I, Schneider M, Bertele D, Debatin KM, Fulda S (2011). Targeting aberrant PI3K/Akt activation by PI103 restores sensitivity to TRAIL-induced apoptosis in neuroblastoma. Clin Cancer Res.

[40] Segerström L, Baryawno N, Sveinbjörnsson B, Wickström M, Elfman L, Kogner P, Johnsen JI (2011). Effects of small molecule inhibitors of PI3K/Akt/mTOR signaling on neuroblastoma growth in vitro and in vivo. Int J Cancer.

[41] Subramonian D, Phanhthilath N, Rinehardt H, Flynn S, Huo Y, Zhang J, Messer K, Mo Q, Huang S, Lesperance J, Zage PE (2020). Regorafenib is effective against neuroblastoma in vitro and in vivo and inhibits the RAS/MAPK, PI3K/Akt/mTOR and Fos/Jun pathways. Br J Cancer.

[42] Fruman DA, Chiu H, Hopkins BD, Bagrodia S, Cantley LC, Abraham RT (2017). The PI3K pathway in human disease. Cell.

