# Supplementary figures and images for: Matrine induces autophagy in human neuroblastoma cells via blocking the AKT-mTOR pathway
Source: Med Oncol. 2022 Aug 16;39(11):167. doi: 10.1007/s12032-022-01762-4 (PMC9381455; doi:10.1007/s12032-022-01762-4)

## Slide 1
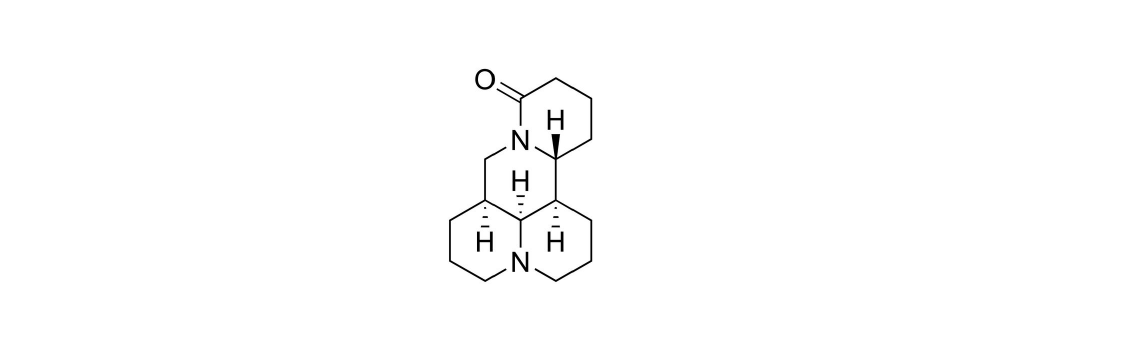

Supplement: Supplementary file 1 — Supplementary file1 Fig. S1 The structure of matrine. Formula: C15H24N2O, Molecular Weight: 248.36, CAS No. 519-02-8 (PPT 137 kb) [file 12032_2022_1762_MOESM1_ESM.ppt]
