# Supplementary material for: Matrine induces autophagy in human neuroblastoma cells via blocking the AKT-mTOR pathway
Source: Med Oncol. 2022 Aug 16;39(11):167. doi: 10.1007/s12032-022-01762-4 (PMC9381455; doi:10.1007/s12032-022-01762-4)
Supplement: Supplementary file 2 — Supplementary file2 Fig. S2 Effects of matrine on the cell cycle in human NB cells. Representative flow cytometry analysis of the cell cycle in SK-N-AS and SK-N-DZ cells treated with DMSO or matrine for 48 h (PPT 192 kb) [file 12032_2022_1762_MOESM2_ESM.ppt]

## Slide 1
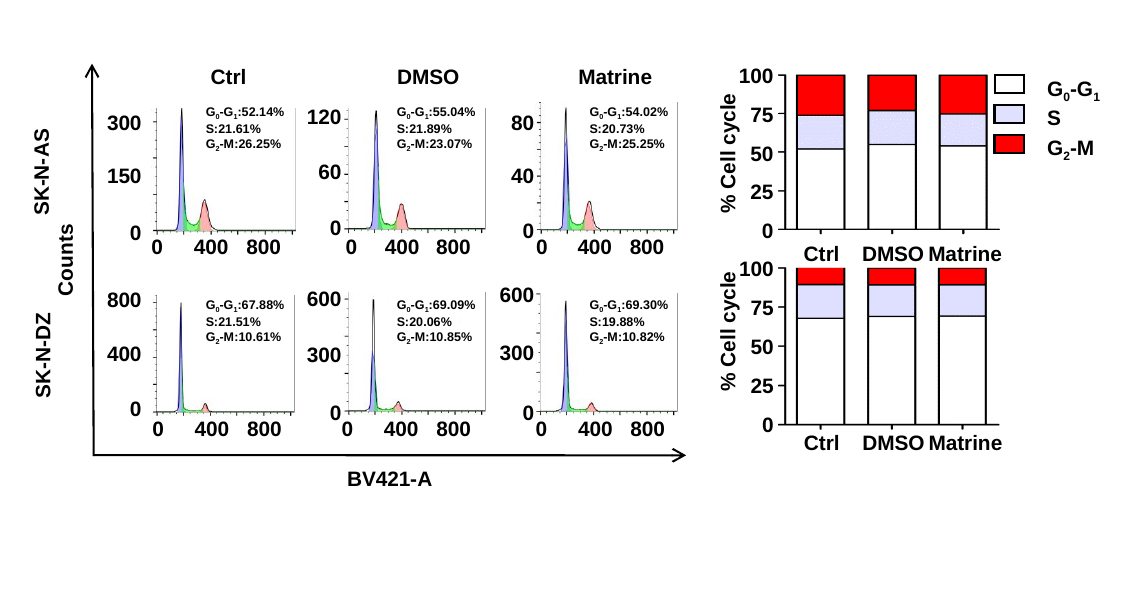

100
% Cell cycle
75
50
25
0
Ctrl
DMSO
Matrine
Ctrl
DMSO
Matrine
G0-G1
S
G2-M
G0-G1:52.14%
S:21.61%
G2-M:26.25%
120
60
0
G0-G1:55.04%
S:21.89%
G2-M:23.07%
G0-G1:54.02%
S:20.73%
G2-M:25.25%
300
150
0
80
40
0
SK-N-AS
Counts
0
400
800
0
400
800
0
400
800
100
% Cell cycle
75
50
25
0
Ctrl
DMSO
Matrine
600
300
0
600
300
0
800
400
0
G0-G1:67.88%
S:21.51%
G2-M:10.61%
G0-G1:69.09%
S:20.06%
G2-M:10.85%
G0-G1:69.30%
S:19.88%
G2-M:10.82%
SK-N-DZ
0
400
800
0
400
800
0
400
800
BV421-A
