# Supplementary material for: Matrine induces autophagy in human neuroblastoma cells via blocking the AKT-mTOR pathway
Source: Med Oncol. 2022 Aug 16;39(11):167. doi: 10.1007/s12032-022-01762-4 (PMC9381455; doi:10.1007/s12032-022-01762-4)
Supplement: Supplementary file 3 — Supplementary file3 Fig. S3 3-methyladenine rescued the short-term cell viability from matrine-induced antiproliferative activity in NB cells. The short-term cell viability of SK-N-AS and SK-N-DZ cells treated with DMSO or matrine alone or in combination with 3-methyladenine were evaluated by CCK-8 assay. 3-MA, 3-methyladenine. **P<0.01, and ***P<0.001 vs. control (PPT 164 kb) [file 12032_2022_1762_MOESM3_ESM.ppt]

## Slide 1
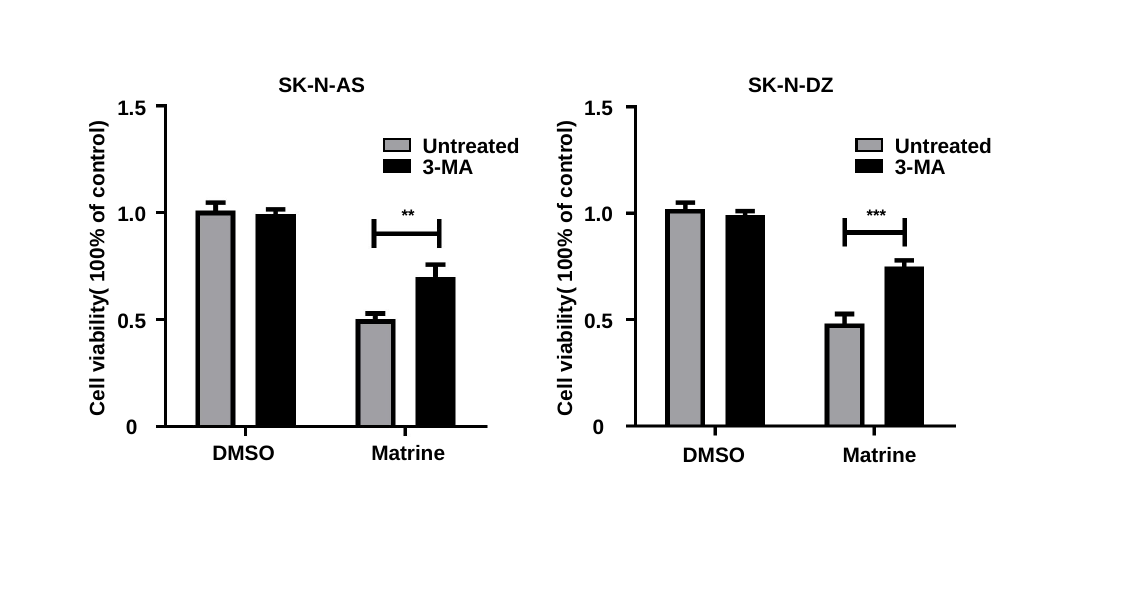

SK-N-AS
SK-N-DZ
1.5
1.5
Cell viability( 100% of control)
Cell viability( 100% of control)
Untreated
3-MA
Untreated
3-MA
1.0
1.0
**
***
0.5
0.5
0
0
DMSO
Matrine
DMSO
Matrine
